# Data Integration–Possibilities of Molecular and Clinical Data Fusion on the Example of Thyroid Cancer Diagnostics

**DOI:** 10.3390/ijms231911880

**Published:** 2022-10-06

**Authors:** Alicja Płuciennik, Aleksander Płaczek, Agata Wilk, Sebastian Student, Małgorzata Oczko-Wojciechowska, Krzysztof Fujarewicz

**Affiliations:** 1 Department of Systems Biology and Engineering, Faculty of Automatic Control, Electronics and Computer Science, Silesian University of Technology, Akademicka 16, 44-100 Gliwice, Poland; 2 Department of Technology Development, Gabos Software Sp z o.o., Mikołowska 100, 40-065 Katowice, Poland; 3 Department of Applied Informatics, Faculty of Automatic Control, Electronics and Computer Science, Silesian University of Technology, Akademicka 16, 44-100 Gliwice, Poland; 4 Department of Biostatistics and Bioinformatics, Maria Sklodowska-Curie National Research Institute of Oncology, Gliwice Branch, Wybrzeze AK 14, 44-100 Gliwice, Poland; 5 Biotechnology Center, Silesian University of Technology, Bolesława Krzywoustego 8, 44-100 Gliwice, Poland; 6 Department of Clinical and Molecular Genetics, Maria Sklodowska-Curie National Research Institute of Oncology, Gliwice Branch, Wybrzeze AK 14, 44-100 Gliwice, Poland

**Keywords:** data integration, biomarkers, bioinformatics, thyroid cancer, data fusion, cancer, classification

## Abstract

(1) Background: The data from independent gene expression sources may be integrated for the purpose of molecular diagnostics of cancer. So far, multiple approaches were described. Here, we investigated the impacts of different data fusion strategies on classification accuracy and feature selection stability, which allow the costs of diagnostic tests to be reduced. (2) Methods: We used molecular features (gene expression) combined with a feature extracted from the independent clinical data describing a patient’s sample. We considered the dependencies between selected features in two data fusion strategies (early fusion and late fusion) compared to classification models based on molecular features only. We compared the best accuracy classification models in terms of the number of features, which is connected to the potential cost reduction of the diagnostic classifier. (3) Results: We show that for thyroid cancer, the extracted clinical feature is correlated with (but not redundant to) the molecular data. The usage of data fusion allows a model to be obtained with similar or even higher classification quality (with a statistically significant accuracy improvement, a *p*-value below 0.05) and with a reduction in molecular dimensionality of the feature space from 15 to 3–8 (depending on the feature selection method). (4) Conclusions: Both strategies give comparable quality results, but the early fusion method provides better feature selection stability.

## 1. Introduction

In cancer diagnostics, many different methods and approaches are used independently. New diagnostic tools are implemented into common use if they are characterized by higher diagnostic abilities (for example, high accuracy or specificity/sensitivity balance) and (possibly) lower costs. Construction of such tools typically begins with massive data obtained from radiology and medical imaging, or high-throughput molecular methods, including microarrays, next generation sequencing, and protein mass spectrometry. These technologies are good sources of data for machine learning (ML); supervised learning and sample type prediction are common techniques applied in diagnostics for a wide range of diseases [1]. Since the quality of classification depends on the quality of features used in the ML model, finding the optimal feature set is a milestone for such research [2].

Designing a diagnostic test from molecular data involves many challenges due to high-dimensionality and (usually) a limited number of samples. During the process of molecular test construction, the sample collection must be split into multiple subsets, such as test set and learning set. The latter must be split again for comparison of learning techniques using cross-validation methods, such as leave-one-out, bootstrap, or k-fold. Consequently, the number of samples available for training the model shrinks, which may influence the dimensionality reduction process if there are multiple intrinsic subgroups already present in the datasets. The molecular features obtained with high throughput techniques showed multiple dependencies. The relations between such a number of features measured with, e.g., correlation, mutual information, or distance measures, are hard to present/interpret in a readable form, and necessitate dimensionality reduction, which may remove clusters of differential features.

One popular method in the context of data integration is feature extraction, which reduces the feature space by creation of new features basing on the existing ones. This is a useful tool to decrease the number of genomic features (as one of the approaches to resolving the ‘curse of dimensionality’ problem) [3]. Some notable methods of feature space reduction for microarrays are principal component analysis (PCA) and partial least squares (PLS) [4]. For other data types, such as text or genomic sequences, the Bayesian methods and Bayesian networks are employed [5].

In order to improve the quality of classification models, data from multiple sources may be used. For the analysis and classification of data from multiple sources, such as -omic, clinical, and others, data integration is a useful practice. Data integration is a broad topic and is linked to data warehousing [6], data migration, and meta-dimensional approach [7]. Methods for combining data from multiple sources may be involved in different stages of research. The early stage covers the preparation of the initial dataset, the intermediate stage includes multiple data manipulations, and the late stage is for combining the final results of previous steps [8]. Thus, despite multiple attempts at formalization, the definition of data integration is fuzzy, and might be summarized as obtaining valuable information from multiple sources.

Data integration may be performed with multiple methods, as described in the literature. Data fusion could be considered a stage of data integration [9], and techniques may be classified according to (1) the relationships between the fused source data, such as complementary data or redundant data [10], (2) the sources of input data [11], and (3) the level of data processing, such as raw data, preprocessed data, or decisions [12]. For the purpose of this paper, we define data fusion as a process of merging two feature sets, regardless if the data are of the same size and comparable/of similar origin (such as merging two sets of microarrays) or not (data from heterogenous sources) [13].

We hypothesize that some clinical data representing the phenotype of a disease may contain complementary data to those of gene expression in terms of features for the classifier, and we investigated the impact of data fusion on the feature selection process. The proper selection of features has become a science of its own and has been widely described, for example in [14]. Based on work by Guyon and Elisseeff [15], features with similar distributions may still improve classification outcomes. We conclude that under careful investigation it is possible to reduce the number of molecular features by using additional variables.

In the present study, we investigated if a molecular classifier may benefit from data fusion resulting in a reduced number of features (lowering the cost of gene expression test) while keeping the accuracy comparable to a molecular test using only gene expression data. Here, we focused on the use of data fusion between high-dimensional gene expression data and low-dimensional clinical data, whose costs are already included in the basic diagnostics process. The inclusion of clinical data in the model may reduce the number of necessary molecular features, decreasing the cost of the whole test. The method we propose allows for assessing the usefulness of fused features and presents the most beneficial feature combination.

We demonstrate the effectiveness of our method using data from thyroid cancer (in which overtreatment is a growing concern). A fine needle aspiration biopsy (FNAB) is the main tool for diagnosis of thyroid nodules [16]. The sample is usually classified according to the Bethesda System for Reporting Thyroid Cytopathology [17,18], which in most cases allows clinicians to distinguish between a benign nodule and malignant one [19]. The results of FNAB might be combined with other diagnostic methods, such as thyroid imaging, reporting, and data system (TI-RADS), to improve the accuracy of the diagnosis [20]. The use of molecular data for classification is beneficial and increases the quality of diagnosis for patients with thyroid nodules, especially in cases of uncertain cytology results, which may account for up to 30% of all biopsies.

References [21,22,23]. Gene expression, which might be determined from FNAB material, has been used in multiple studies [24,25], but some difficulties remain, including the lower availability of molecular tests in low-income countries or insufficient validation on different populations [26,27].

Molecular markers, as shown in the studies presented above, are promising for cancer diagnostics. A reduction in the number of molecular markers without any significant losses in accuracy will lower the costs of the test and make it more available for a wider group of patients. In this study, we compare the different integration strategies using sets of microarray gene expression profiles and sets of the most common clinical factors claimed to be risk indicators (or valuables for patient prognosis). We compare the results of data fusion for two popular feature selection methods in terms of classification accuracy and feature ranking stability. Because the aim of genomic feature selection is to find the optimal set of molecular markers, whose expressions may be measured with different methods, such as RT-qPCR (reverse transcription quantitative real-time polymerase chain reaction), the presented study excludes the use of feature extraction techniques for molecular data.

## 2. Results

### 2.1. Clinical Feature Extraction

**Figure 1:**
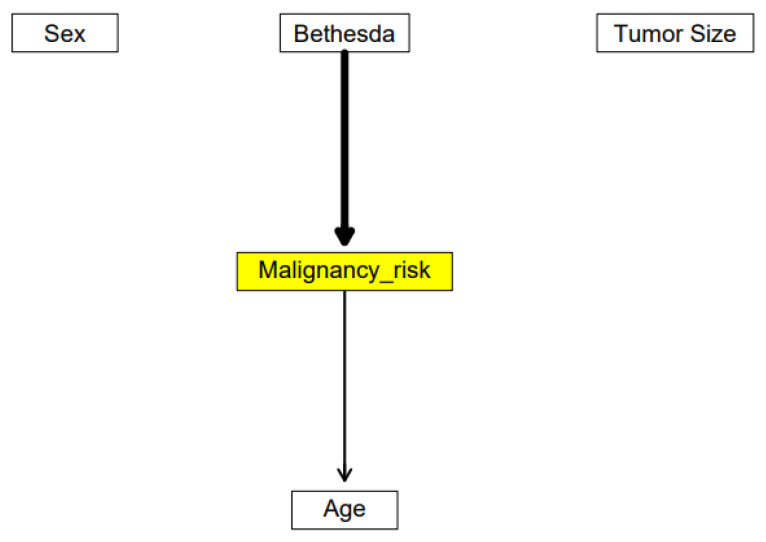
The estimated Bayesian network structure. The yellow node represents the outcome—the Malignancy_risk—the source of our new variable. The arrows represent strong connections between nodes; however, their meanings may be interpreted as: the Bethesda influence on risk; age should be considered as an indicator for an in-depth analysis toward thyroid cancer. Both nodes belong to the Markov-blanket of Malignancy_risk and should be included when performing inference on a given node.

Instead of using the clinical data in raw form, we integrated all non-genomic data into one model to obtain one numeric value—the probability of a nodule’s malignancy. We used a Bayesian network; the dependent variable is represented by the node we called Risk. The graph for the created network is presented in Figure 1.

The graph presents the connections that occur most frequently between variables. Malignancy risk is independent from sex and tumor size but we have found connections between malignancy risk and both age and Bethesda. The strength of the connection between Bethesda and malignancy is 10 times stronger than between age and malignancy. This should be interpreted as follows: the sex and size of the lesions show nothing special about the malignancy risk, but knowing the patient’s age, we can speculate about the probability of the malignant type of nodules. The risk of having a malignant nodule increases with age. The lack of connections between sex and age may be a result of a low number of samples and internal bootstrapping.

#### Data Dependencies

We used two sets of molecular features previously published as markers for genomic classifiers, which we called Microarray_163 and Microarray_40. We estimated the similarity between features, because we considered that they may influence the stability of feature selection. We calculated Spearman’s correlation for each gene pair in genomic feature sets and for each gene and malignancy risk. For those pairs, we also estimated mutual information.

**Figure 2:**
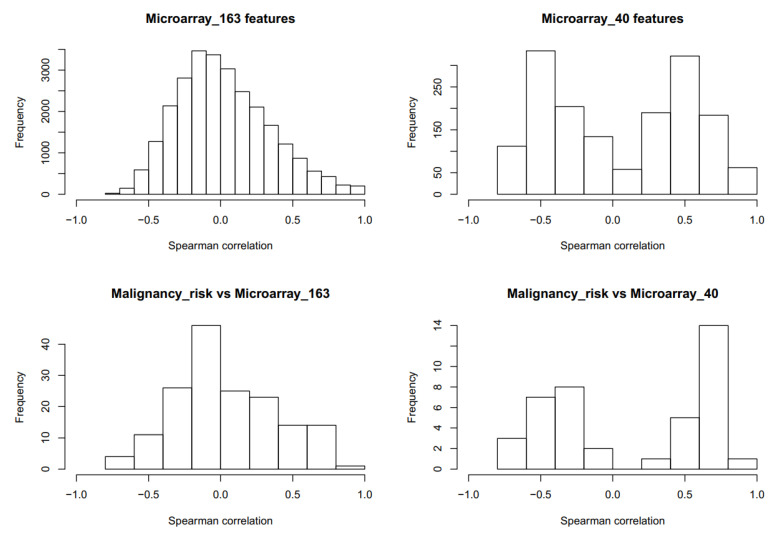
Histograms of correlation coefficients within the analyzed sets of molecular features (gene expression) and molecular features and malignancy risks for given samples. Please note that the number of high correlation coefficients between malignancy risk and genomic features is similar in both datasets.

We checked the relations between each feature pair in the given genomic feature sets. The dataset differences (e.g., in sizes and histograms of Spearman’s correlation values) are presented in Figure 2. For the large feature set (163 features), the distribution was similar to the Gaussian distribution. Most feature pairs showed very weak correlation or a lack thereof. In this feature set, only a small number of pairs showed high correlation coefficients; additionally, the correlation was mainly positive. The smaller dataset showed a different distribution; here, the majority of pairs showed moderate (but both positive and negative) correlation values (bimodal distribution).

Then, we compared the malignancy risk with each molecular feature for both molecular feature sets. For the Microarray_163 feature set and clinical risk, the correlation coefficients were in the range between moderate negative correlation and moderate positive correlation. For this feature set, most pairs had correlation coefficient close to 0. The smaller dataset showed bimodal distributions for molecular feature correlations and molecular features versus malignancy risk. Eventually, the number of moderate or highly correlated pairs of molecular featured and malignancy risks was comparable in both datasets

**Figure 3:**
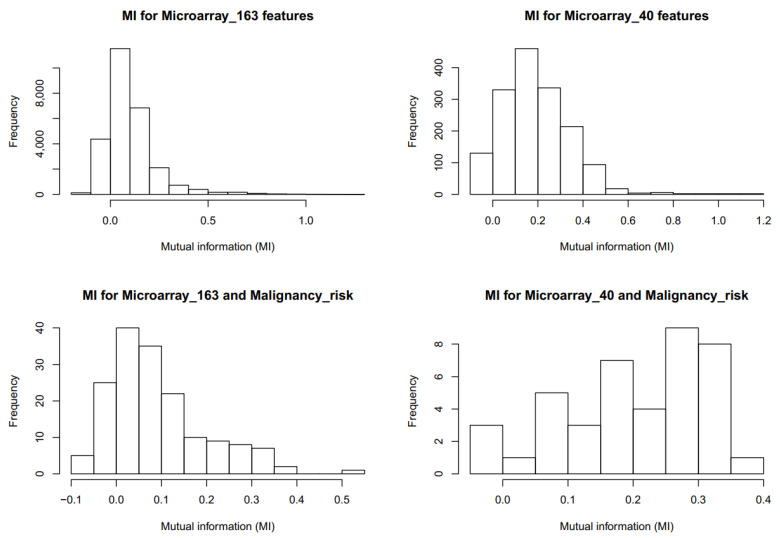
The histograms of mutual information between each pair of genomic features and between Malignancy_risk and genomic features.

We calculated the mutual information (MI) between the molecular features (histograms are presented in Figure 3A) and between molecular features and malignancy risk (histograms in Figure 3B). The MI values for molecular features mostly showed independency between features. Only a few pairs of molecular features showed high values of MI (over 0.6). The MI between the malignancy risk and molecular features was rather low. For the Microarray_40 feature set, there were no pairs with MIs higher than 0.4. For the Microarray_163 feature set, there was one risk-gene pair with MI about 0.5. As presented, the mutual information showed that, in comparison to Spearman’s correlation coefficient, the dependencies between risk and features were rather low. This means that features were not redundant, but they were not totally independent.

### 2.2. Classification Accuracy

For feature selection, we used two popular filter methods, the Wilcoxon test and the ReliefF algorithm, often used for genomic feature selection [28]. In this study, we present results for a wide range of models (for increasing the number of selected features). We investigated up to 40 features selected with those methods (41 for the late fusion strategy) because we cut the number of features to the smallest feature set. We compared the results for each model (all bootstrap iterations) between the strategies (early fusion and late fusion versus no-fusion model) with a Wilcoxon–Mann–Whitney test.

**Figure 4:**
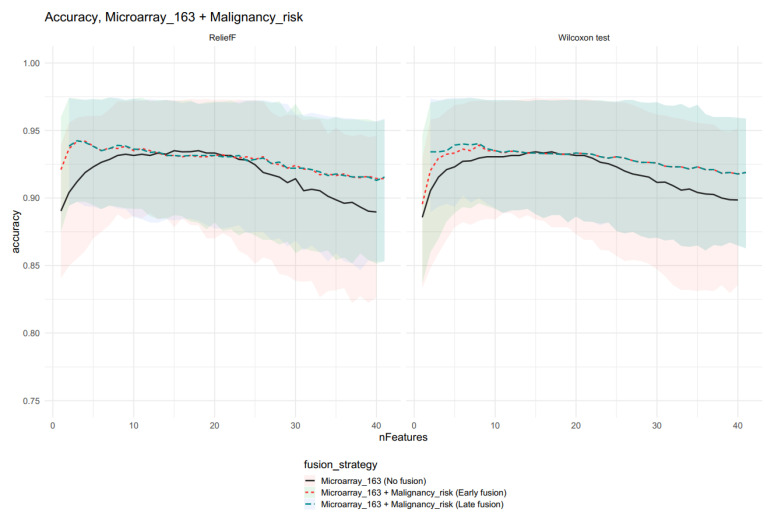
Comparison of the different fusion strategy model accuracies with confidence intervals for the Microarray_163 feature set and Malignancy risk (Risk). Please note that for the ReliefF method, the data fusion strategies show similar accuracies.

We calculated median bootstrap accuracy for all models. The accuracy for malignancy risk was 0.92 (CI: 0.88–0.96). Then we compared the reference models (molecular features only) and models with data fusion. We tested a wide range of selected features for each feature selection method. For the Microarray_163 feature set, we tested a whole range of features, but the accuracy decreased with the increasing number of predictors, so we analyzed only the range of 1–41 features. For each reference model, we observed a maximum accuracy for models with 15–16 features. For models with higher numbers of features, the accuracy slowly decreased. For both feature sets, the models with fusion had higher accuracies than the reference model for the small number of features. The results for models based on Microarray_163 are presented in Figure 4.

**Figure 5:**
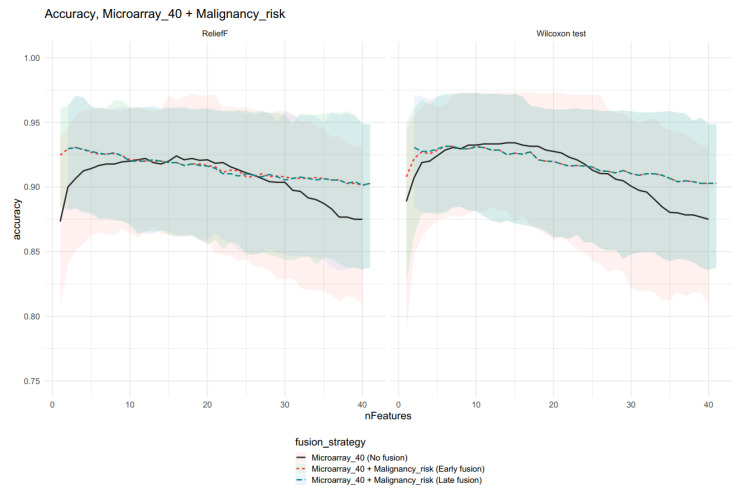
Comparison of different fusion strategy model accuracies with 95% confidence intervals for the Microarray_40 feature set and Malignancy risk (risk). Please note that for the ReliefF feature selection, the method resulted in similar accuracies for models with 2–15 features. Moreover, this feature selection method resulted in a lower accuracy for the no fusion model than the Wilcoxon test method.

The differences between feature selection algorithms are weak, but models with data fusions have higher accuracies than the best reference model with 15 features. Models with features form Microarray_40 are presented on Figure 5, and in this case, the Wilcoxon test had a slightly higher accuracy; the difference between the models with fusion and the reference model were negligeable.

For the Wilcoxon feature selection, the accuracy results were slightly higher for the late fusion strategy, but the confidence intervals overlapped.

These results suggest that data fusion allows for increased accuracy with a reduction of the necessary features. In this case, late fusion seems to be more beneficial. We compared the best reference model with the best models of fusion and the p-values, presented in Table 1.

We checked if the similarity of the early fusion to the late fusion strategy meant that the malignancy risk was selected as often as in late fusion (in each model). For such a case, the model’s accuracy and feature selection stability would be similar for both data fusion strategies. The exact values of the *p*-values and the differences between the model with fusion and the expression-only model for the nFeatures range of 1–15 are presented in the Supplementary Materials (Table S1 and Table S2, respectively, for Microarray_163 and Microarray_40 features sets).

### 2.3. Stability of Feature Selection

While the performance of a model (in terms of classification accuracy) is crucial for its applicability, molecular markers can also provide valuable insight through pathway and functional analyses. Therefore, feature selection should also be evaluated for its consistency. We used the Kuncheva stability index [29], which accounts for selection reproducibility; its constraints are not dependent on the number of features or repetitions [30]. We focused on the Kuncheva index because it allows for comparison of multiple pairs of features, seems to be simple, and is suitable for bootstrap sampling.

As expected, slight differences were observed. The early fusion strategy seemed to have higher Kuncheva index scores than the reference model and the late fusion strategy. The unexpected result was that the early data fusion strategy and ReliefF feature selection helped increase the feature selection stability. We analyzed the feature rankings, and indeed, the selected feature lists were shorter for the early data fusion strategy.

**Figure 6:**
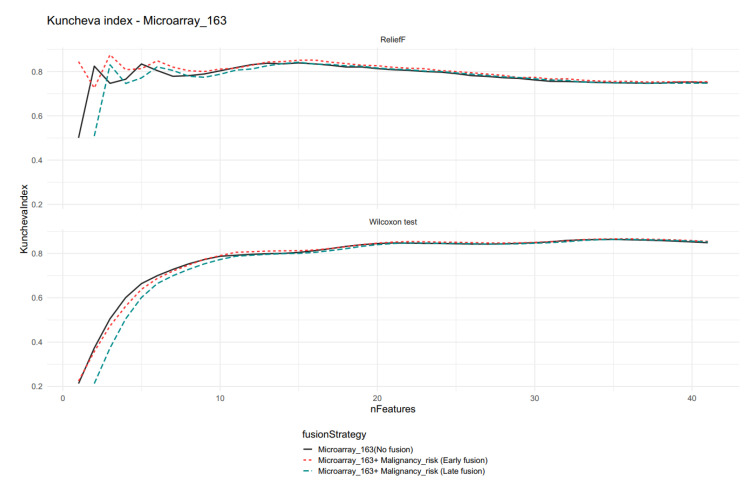
Comparison of the Kuncheva index for different data fusion strategies for Microarray 163 and Malignancy_risk features. Note the difference between the two feature selection methods for models with low feature numbers (nFeatures).

For the Microarray_163 feature set, the Kuncheva index is presented in Figure 6 for models containing 40 (41 for the late fusion strategy) features, and its values converged for all methods, which was caused by the similarity of rankings and low influence of additional features for the late data fusion strategy.

**Figure 7:**
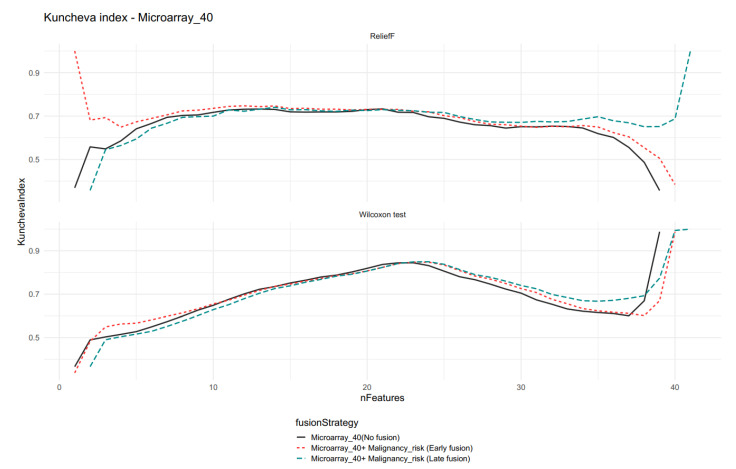
Comparison of the Kuncheva index for different data fusion strategies for Microarray 40 and Malignancy_risk features. Note the difference between the two feature selection methods for models with a low number of features (nFeatures). For the nFeatures close to the maximum number of features, the stability obtained the higher value and rose to its limit.

The Kuncheva indexes for the Microarray_40 feature sets showed higher differences as the feature selections reached the maximum of the available features. The maximum values of each model reached the maximum stability (all features were selected) and are omitted in Figure 7.

For a low number of features (where accuracy was highest), early fusion increased the stability of the feature selection for both ReliefF and Wilcoxon methods. The malignancy risk was selected, appeared on the top positions of the feature ranking, and enhanced the stability for the selection of molecular features. Higher stability is desired for feature selection, making the early fusion strategy a better solution for a given dataset, because this method produces a similar (or slightly lower) quality performance.

## 3. Discussion

Summarizing this study, we compared two data fusion strategies for the two types of feature sets: high-dimensional molecular data and clinical data represented by one extracted feature—malignancy risk. We used two popular ranking-based feature selection methods—ReliefF and the Wilcoxon test. We observed the influence on classification accuracy (using bootstrap) and the stability of feature selection.

We added one continuous feature to the preselected subset of features and obtained a higher accuracy than the best subsets of the given molecular features and the malignancy risk itself.

For the analyzed dataset, the accuracies of both methods were comparable, but they differed in the stability of feature selection. We should highlight that the comparison of data fusion strategies is far more informative than the analysis of feature dependencies. We observed some correlations between features but mutual information showed that the dependencies between malignancy risk and gene expression were rather weak. The analysis of dependencies between features may influence the feature selection method so a preliminary analysis of the data is still an important step in the process of feature selection. The dependencies between features are important to choose the classification method. Some models based on, e.g., logistic regression, may not work well when features are redundant. In this study, we used SVM with a linear kernel but the influence on classification accuracy may be different for other classification methods.

The Kuncheva index is a very sensitive tool to investigate the differences between fusion strategies, especially for the number of features close to the minimum and maximum available features. According to [30], this index contains the correction for the intersection by the chance. There are many other methods used to assess the stability of feature selection [31], some of them also consider the position in feature ranking or monotonicity. However, the usability of those methods requires further investigation. The Kuncheva index is easy to implement and it is very intuitive to use with bootstrap cross-validation. In this study, we compared our data fusion models by the number of features, which allowed us to observe the effects of added clinical features on the feature selection stability. For models with late fusion, we expect that the stability will be higher for models with a large number of features because we will always add the same feature for each model. For early fusion, we expected lower stability, because the added feature had similar distributions of dependencies as the molecular features itself. The higher stability for early fusion means that the Malignancy_risk is somehow similar to the best genomic features, and together they provide a better separation of two classes.

The data fusion strategies presented here resulted in the increase in the maximum classification quality or kept it on a similar level as the best reference model, but with a considerable reduction in the number of predictors. However, the effect of data fusion was relevant to the interrelation between features. The stability index and comparison of the two data fusion strategies showed the influence of the added features. The effect of adding one feature in the fusion process was easy to detect in the feature rankings, but for multiple models and multiple features, it became a tedious task.

The dependencies between features in the data fusion influenced the classification’s accuracy; however, a feature not correlated enough with the classifier outcome (or other features) may have a negative influence on the model’s quality. The data fusion of the other data types may improve the classification of the specimen, and cause the features collected for the lesions from different diagnostic procedures to set different points of view on the specimen. The features used for fusion may be related (such as in genotype—expression—phenotype relation); for example, the cytological picture of the lesion is caused by the changes in the molecular mechanisms in the cells. The data fusion may extend the list of significant features with new molecular markers of cancer.

In this study, the Wilcoxon test achieved slightly higher stability but the ReliefF method seemed to stabilize the selection after fusion of genomic features and malignancy risk. The steps to take following this research would be the comparison of more feature selection algorithms. We expected some differences; although they are both ranking methods, the ReliefF algorithm is more complex and uses internal shuffling of the sample and feature subsets in calculations of the feature weights while the ranking based on the Wilcoxon test *p*-values are made from independent tests for each feature (needing *p*-value adjustments). For the other real data, use of the preselection algorithm may be necessary (here, we used preselected—but large—feature sets).

In the present study, the data fusions were tested on one type of variable (continuous variables) and the simple combination of fusion and the feature selection, which showed a statistically significant accuracy improvement for models with few features in comparison to the homogenous feature set.

The methods presented are not limited to thyroid cancer, but they need further investigations. We see a possibility of using the data fusion of molecular data with results in other diagnostic procedures, such as in the analysis of mutations, checking immunohistochemical markers, and imaging techniques, such as tomography, ultrasounds, magnetic resonance imaging, positron emission tomography, or others. Some of them may use advanced methods of feature extraction (especially imaging) but the methods are well described in the literature and the data fusion may be involved in studies after preprocessing of the features. The success of data fusion depends on the relationship between features. By the present study, the presence of a strong correlation and high mutual information with a few molecular features is beneficial to reduce the number of features. In the case of data fusion with the features represented (with ordinal or categorical variables), some other measures of similarities may be used, such as other implementations of mutual information (using only discrete values) or the Kruskal–Wallis test for mixtures of continuous and discrete variables. The method of data fusion may have some limitations in the case of cancer heterogeneity; however, more excessive research should be performed in this field.

## 4. Materials and Methods

### 4.1. Dataset

The thyroid dataset consisted of 200 samples provided by Maria Sklodowska-Curie National Research Institute of Oncology Gliwice Branch. Biological materials for the microarray experiments were extracted from the biopsy of patients’ thyroid nodules. To each microarray, a label (acquired from histopathology studies) was assigned, indicating cancer (77 samples) or benign lesions (123 samples). The samples were assessed with a cytopathological test and fell into Bethesda categories II (benign) to VI (malignant), but the majority of samples were from III to V. Gene expression data was obtained using Gene Chip Affymetrix human transcriptome 2.0 arrays (HTA 2.0, Santa Clara, CA, USA). GeneChip WT Pico Reagent Kit (Santa Clara, CA, USA) were used for microarray preparation. All reactions including DNA target preparation, target hybridization, fluidics setup and array scanning were performed according to the user guide P/N 703262 rev 4. Skanning of the arrays were performed in GC30007G scanner.

The preprocessing of the raw microarray dataset was conducted using the Aroma tool implemented in the R/Bioconductor environment [32]. For background corrections, we used the Robust Multichip Analysis (RMA); the normalization process was conducted using the quantile method. The summarization process was conducted using the log-additive model and median polish estimator. To map the probes on the dataset arrays to ENTREZ genes, we used a custom chip definition file (CDF) downloaded from the Brainarray website [33]. The control probes were removed in order to preserve only the values of gene expressions in the dataset. After preprocessing the microarrays, 32,500 normalized expression values were left for each sample.

To avoid problems with continuous data resulting from unknown types of distributions of clinical features [34], we discretized the data using an expert’s knowledge. Intervals for age were set to less than 20 years, (20, 45], (45, 60], and more than 60 years, which correspond to the clinical factors of increased malignancy risks in a thyroid lesion [35]. Intervals for tumor sizes were set according to the thresholds used in cytology examinations, starting from less than 0.5 cm (XS), (0.5, 1] (S), (1, 1.5] (M), (1.5, 2.5] (L), (2.5, 4] (XL), and more than 4 cm (XXL) [36]. Remaining features, such as sex, and cytological grade, were already discrete.

The correlations and mutual information were calculated using R. Mutual information was calculated using Kraskov’s method [37], using R implementation in the parmigene package [38].

### 4.2. Clinical Feature Extraction

In this paper, we used the Bayesian framework as part of a supervised analysis. As described in [39], the network’s structure was learned on the set of clinical data gathered from patients (from whom we collected the molecular data). The dependent variable was represented by the node we called risk. We estimated the parameters of the network, which resulted in a conditional probability table (CPT) for each node based on a combination of all possible values of ancestor nodes. To avoid the zero probabilities caused by zero counts for particular parent values, we used the Bayesian estimation method to learn the parameters. We used the R package bnlearn [40] from the CRAN repository. ‘Network’ was learned using the score-based method Tabu Search to discover a completely directed network with Akaike information criterion (AIC). We used the method of likelihood weighting, which is a sampling technique where the variables are sampled in the order defined by a network to estimate the sample probability of not being rejected. To avoid an information leak, we learned the structure of the network and estimated the parameters using k-fold cross-validation (k = 10). To obtain probabilities of events (the particular values of dependent variables), one should query the network with given evidence. The results of the query were calculated using the Markov-blanket (parents, children, and all other nodes sharing a child), which is the minimal set of nodes necessary to obtain faithful results. For each patient’s clinical data, as evidence, we queried the network and calculated the value of malignancy probability; we called this new feature a malignancy risk. These values were used for further data fusion methods with molecular data.

### 4.3. Feature Sets

The analysis was based on two gene sets (indicated as molecular markers for thyroid cancer). The first set published by Fujarewicz et al. [25] contained 40 features to distinguish between benign nodules and the most common thyroid cancer type—the papillary thyroid carcinoma. The second one with 163 features was published by Alexander et al.’s [41] extended scope for other malignant thyroid conditions (such as other types of thyroid carcinoma or the most common metastases). Those two gene sets shared seven features.

From each patient, the clinicians collected the data they deemed necessary to make decisions about the patient’s treatment, such as age, sex, tumor size, and the grade of disease progress assessed by the specialist. The summary of the used feature sets is presented in Table 2.

### 4.4. Data Fusion

We compared the accuracies of the supervised learning models, varying in terms of parameters: (1) applied data fusion strategy, (2) feature selection method. As a reference, we used separate models for each feature subset (clinical models and molecular models).

**Figure 8:**
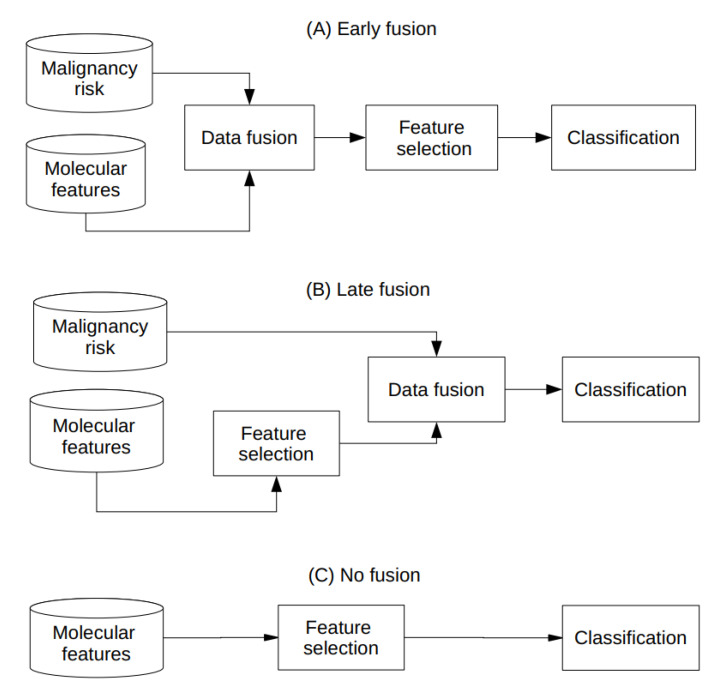
The difference between early fusion (**A**) and late fusion (**B**) with comparison to no fusion (**C**).

The clinical data were fused with genomic data by one of the following strategies: (1) data fusion performed after genomic feature selection (late fusion), (2) data fusion performed before feature selection (early fusion). Late fusion allowed simply adding all variables to the preselected genomic features and then we could measure the effect on classification accuracy in comparison to the one source of the data—the gene expression data only. Early fusion allowed combining information and selecting only the meaningful features from both data sources. In order to unify the data obtained by the classifier function for each strategy, the z-score normalization was performed as follows: (1) the whole dataset was normalized after fusion (and before classification) for late fusion; (2) the whole dataset was normalized before feature selection for early fusion. All fusion strategies are summarized in Figure 8.

### 4.5. Feature Selection and Classification

To validate the classification results, we used the bootstrap technique with 500 iterations. The stages of fusion, feature selection, normalization, and classification were inside the bootstrap loop to avoid an information leak. For each bootstrap iteration, we calculated accuracy based on a test set. Median values for the models based on all bootstrap iterations are presented in the tables and figures. Calculations were performed using SPICY project and the R environment [42].

The feature selection methods were implemented in the SPICY package. The Wilcoxon test features were ranked based on corrected *p*-values (Benjamini–Hochberg procedure), and the first N features with the smallest values were selected for classification. For the ReliefF feature selection, we implemented the CORElearn package [43].

Classification accuracy is a common metric for the evaluation of the feature selection methods [44]. In the present study, we used the SVM classifier (linear kernel) to evaluate the feature selection methods and fusion strategy.

### 4.6. Stability of Feature Selection

The index is calculated as follows. For a sequence *S* = {*S*_1_, *S*_2_, …, *S _K_*} containing sets of features, obtained by performing feature selection on *K* different samplings of a dataset, the Kuncheva stability index *κ* (*S*) is defined as:

(1) $$K\left(S\right)=\frac{2}{K\left(K-1\right)}\sum_{i=1}^{K-1}\sum_{j=i+1}^{K}\frac{\left|S_{i}\cap S_{j}\right|n-k^{2}}{k\left(n-k\right)},$$

where *k* is the number of selected features (equal for all sets in *S*), *n* is the number of all features, and |.| denotes the set cardinality. The high stability of feature selection ensures that all markers are indeed relevant.

We compared the stability between early and late fusion. For early data fusion method, the use of the stability index was intuitive, but for late data fusion, where the fusion came after the feature selection, we gathered all features involved in classification for each bootstrap model (as *S _K_*). This approach allows comparing stability results for general purposes and use with any other combination of data fusion. Thus, the expected results of the comparisons of both fusion strategies were the higher accuracy for late fusion, because at least one feature appeared in each list of features before classification.

## Figures and Tables

**Table 1:** Summary of data fusion models with the highest accuracies. Notice that early fusion had a higher stability, and the biggest differences were for the ReliefF feature selection method.

Data	Strategy	Feature Selection	Accuracy (Median)	nFeatures	Kuncheva Index
Microarray_163 + Malignancy_risk	Early Fusion	ReliefF	0.942	3	0.876
Microarray_163 + Malignancy_risk	Late Fusion	ReliefF	0.942	3	0.753
Microarray_163 + Malignancy_risk	Early Fusion	Wilcoxon	0.939	8	0.747
Microarray_163 + Malignancy_risk	Late Fusion	Wilcoxon	0.940	8	0.753
Microarray_40 + Malignancy_risk	Early Fusion	ReliefF	0.931	3	0.693
Microarray_40 + Malignancy_risk	Late Fusion	ReliefF	0.931	3	0.564
Microarray_40 + Malignancy_risk	Early Fusion	Wilcoxon	0.932	6	0.582
Microarray_40 + Malignancy_risk	Late Fusion	Wilcoxon	0.932	6	0.552

**Table 2:** The summary of feature sets used in the study.

Dataset	Feature Set	Number of Features	Characteristics
Microarray_40	Expression of genes listed in Fujarewicz et al.	40	Binomial distribution of features’ correlation
Microarray_163	Expression of genes listed in Alexander et al.	163	Normal distribution of features’ correlation
Malignancy_risk	Extracted feature with method Płaczek et al.	1	Continuous variable in range 0–1

## Data Availability

The data presented in this study are available upon request from the corresponding author. The data are not publicly available due to pending patents and a related publication based on the extended dataset.

## Supplementary Materials

The following supporting information can be downloaded at: https://www.mdpi.com/article/10.3390/ijms231911880/s1.
