# Supplementary material for: Data Integration–Possibilities of Molecular and Clinical Data Fusion on the Example of Thyroid Cancer Diagnostics
Source: Int J Mol Sci. 2022 Oct 6;23(19):11880. doi: 10.3390/ijms231911880 (PMC9569592; doi:10.3390/ijms231911880)
Supplement: Supplementary file 1 [file ijms-23-11880-s001.zip › ijms-1875226-supplementary.pdf]

Table S1. *The comparison of the fusion models with the no fusion model with the highest median accuracy for the Microarry_163 genomic feature set. The sign represents the difference between median bootstrap accuracy of data fusion model and median bootstrap accuracy of best no fusion model.*

Feature set: Microarray_163,
Null hypothesis: medians are equal

**Feature selection**	ReliefF		ReliefF		Wilcoxon		Wilcoxon	
**Data fusion**	Early fusion		Late fusion		Early fusion		Late fusion	
**nFeatures**	sign	p-value	sign	p-value	sign	p-value	sign	p-value
**1**	-	**<0.001**	NA	NA	-	**<0.001**	NA	NA
**2**	+	**0.015**	+	**0.015**	-	**<0.001**	-	0.642
**3**	+	**<0.001**	+	**<0.001**	-	**<0.001**	-	0.489
**4**	+	**<0.001**	+	**0.001**	-	0.196	+	0.064
**5**	+	**0.002**	+	**0.016**	-	0.946	+	**0.005**
**6**	-	0.115	-	0.357	+	0.129	+	**0.001**
**7**	+	**0.007**	+	**0.001**	+	0.091	+	**0.005**
**8**	+	**0.016**	+	**0.004**	+	**0.003**	+	**<0.001**
**9**	+	**0.004**	+	**0.019**	+	0.09	+	**0.026**
**10**	-	0.147	+	0.053	+	0.326	+	0.234
**11**	+	0.157	+	0.198	-	0.977	-	0.972
**12**	-	0.672	-	0.655	+	0.869	+	0.858
**13**	-	0.425	-	0.569	-	0.981	-	0.972
**14**	-	0.179	-	0.101	-	0.946	-	0.901
**15**	-	0.056	-	**0.027**	-	0.693	-	0.722

Table S2. *Comparison of the fusion models with the no fusion model with the highest median accuracy for the Microarry_40 genomic feature set. The sign represents the difference between median bootstrap accuracy of data fusion model and median bootstrap accuracy of best no fusion model.*

Feature set: Microarray_40,
Null hypothesis: medians are equal

**Feature selection**	ReliefF		ReliefF		Wilcoxon		Wilcoxon	
**Data fusion**	Early fusion		Late fusion		Early fusion		Late fusion	
**nFeatures**	sign	p-value	sign	p-value	sign	p-value	sign	p-value
**1**	+	**0.043**	NA	NA	-	**<0.001**	NA	NA
**2**	+	**<0.001**	+	**<0.001**	-	**<0.001**	-	**0.048**
**3**	+	**<0.001**	+	**<0.001**	-	**<0.001**	-	**<0.001**
**4**	+	**<0.001**	+	**<0.001**	-	**<0.001**	-	**0.002**

**5**	+	**0.002**	+	**0.003**	-	**0.005**	-	**0.018**
**6**	+	0.055	+	0.099	-	0.155	-	0.419
**7**	+	**0.041**	+	0.117	-	0.092	-	0.134
**8**	+	0.072	+	**0.047**	-	0.216	-	0.295
**9**	+	0.136	-	0.495	-	0.135	-	0.145
**10**	-	0.856	-	0.72	-	0.208	-	0.245
**11**	-	0.368	-	0.147	-	0.065	-	0.08
**12**	-	0.439	-	0.195	-	**0.002**	-	**0.003**
**13**	-	0.608	-	0.19	-	**<0.001**	-	**<0.001**
**14**	-	0.495	-	0.482	-	**<0.001**	-	**<0.001**
**15**	-	**0.039**	-	**0.038**	-	**<0.001**	-	**<0.001**
